# Relative seismic velocity variations correlate with deformation at Kīlauea volcano

**DOI:** 10.1126/sciadv.1700219

**Published:** 2017-06-28

**Authors:** Clare Donaldson, Corentin Caudron, Robert G. Green, Weston A. Thelen, Robert S. White

**Affiliations:** 1 Bullard Laboratories, Department of Earth Sciences, University of Cambridge, Madingley Road, Cambridge CB3 0EZ, UK.; 2 U.S. Geological Survey, Cascades Volcano Observatory, Vancouver, WA 98661, USA.

**Keywords:** Seismic noise interferometry, volcano monitoring, volcano seismology, seismic noise-based methods, volcano deformation

## Abstract

Seismic noise interferometry allows the continuous and real-time measurement of relative seismic velocity through a volcanic edifice. Because seismic velocity is sensitive to the pressurization state of the system, this method is an exciting new monitoring tool at active volcanoes. Despite the potential of this tool, no studies have yet comprehensively compared velocity to other geophysical observables on a short-term time scale at a volcano over a significant length of time. We use volcanic tremor (~0.3 to 1.0 Hz) at Kīlauea as a passive source for interferometry to measure relative velocity changes with time. By cross-correlating the vertical component of day-long seismic records between ~230 station pairs, we extract coherent and temporally consistent coda wave signals with time lags of up to 120 s. Our resulting time series of relative velocity shows a remarkable correlation between relative velocity and the radial tilt record measured at Kīlauea summit, consistently correlating on a time scale of days to weeks for almost the entire study period (June 2011 to November 2015). As the summit continually deforms in deflation-inflation events, the velocity decreases and increases, respectively. Modeling of strain at Kīlauea suggests that, during inflation of the shallow magma reservoir (1 to 2 km below the surface), most of the edifice is dominated by compression—hence closing cracks and producing faster velocities—and vice versa. The excellent correlation between relative velocity and deformation in this study provides an opportunity to understand better the mechanisms causing seismic velocity changes at volcanoes, and therefore realize the potential of passive interferometry as a monitoring tool.

## Teaser

Seismic velocity changes correlate with deformation at Kīlauea volcano, advancing noise interferometry as a monitoring tool.

## INTRODUCTION

### Seismic noise interferometry as a monitoring tool

Measurement of seismic velocity by passive interferometry using seismic noise is a promising monitoring tool at volcanoes (*1*, *2*), potentially being sensitive to magma pressurization and redistribution of melt within a subsurface plumbing system. The continuous nature of seismic noise provides better temporal resolution than earthquake interferometry and means that the technique is not dependent on the occurrence and location of seismicity. Furthermore, some authors (*1*) have postulated that ambient noise may be sensitive to changes at depth that do not deform the volcano surface and hence may be missed by measurements from interferometric synthetic aperture radar (InSAR), Global Positioning System (GPS), and tilt meters. Monitoring successes include detection of precursory eruptive signals of relative velocity at Piton de la Fournaise (*1*, *3*) and mapping of pressurized volcanic fluids in Japan (*4*). Detection of signals due to short-term (months to hours) changes in magma pressurization or transport before eruptions is an exciting result. However, these short-term velocity changes have often been seemingly unrelated to other observables at volcanoes during intereruptive periods and over long periods of time (years). In particular, surface deformation—a traditional indicator of volcano pressurization—has not yet been found to correlate consistently with seismic velocity, except for long-term changes that occur over many years (*5*). This lack of definitive correlation has limited our understanding of the mechanisms causing the velocity changes of the shallow subsurface. Kīlauea is an extremely well-monitored volcano with continuous summit activity since 2008 and clear, repetitive deformation transients. This feature provides an excellent opportunity to compare measurements of deformation to seismic velocity changes and to test the possibility of noise interferometry as a monitoring tool. We studied relative velocity variations in the subsurface at Kīlauea volcano over the period of June 2011 to November 2015 using seismic noise interferometry with volcanic tremor as the passive noise source. Over this 4-year period, we find a remarkably consistent correlation of relative velocity and deformation on time scales of days to weeks.

### Kīlauea volcano

**Fig. 1:**
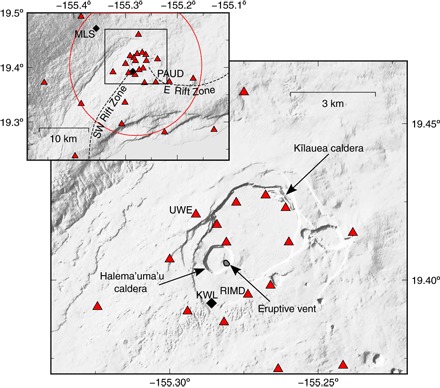
Maps of Kīlauea volcano. Seismic stations are shown as red triangles, and strainmeters are shown as black diamonds. Seismic stations enclosed in the red circle, centered on the eruptive vent, are used to average d*v*/*v*. Seismic instrumentation details are given in Materials and Methods. Tilt data in this study are from a tiltmeter located at UWE (zoomed-in map). The lava lake in the eruptive vent is located within Halema’uma’u caldera, which is itself located within the larger Kīlauea caldera. PAUD, Pauahi, Hawaii Digital; RIMD, Caldera Rim, Hawaii Digital; KWL, Keller Well; MLS, Mauna Loa Strip Road.

An eruptive vent has existed at Kīlauea summit (Fig. 1) in the southeast corner of Halema’uma’u caldera since it opened in March 2008 (*6*). The summit plumbing system is thought to consist of a shallow magma reservoir located on the east side of Halema’uma’u caldera at 1- to 2-km depth below the surface [the Halema’uma’u reservoir (HMMR)] and a larger, deeper reservoir at 3- to 5-km depth below the southern side of Kīlauea caldera (*7*). This magmatic system is linked to the East Rift Zone—in which Puʻu ʻŌʻō vent, 20 km from the summit, has been erupting since 1983—and the Southwest Rift Zone.

Deformation at Kīlauea is the result of both long- and short-term processes. Longer-term deformation includes contributions from volcanic and tectonic sources, such as pressurization of the summit magma system and seaward motion of Kīlauea’s south flank. Deformation at Kīlauea’s summit on the time scale of hours to days is dominated by transient deflation-inflation (DI) events (*8*). They are measured to varying degrees by InSAR, tiltmeters, GPS, and strainmeters and are strongly correlated with changes in surface height of the summit lava lake (*8*–*10*). Inverse modeling of DI events shows that the deformation is generated by pressure transients in the HMMR, located beneath the eastern margin of Halema’uma’u caldera (*8*).

### Seismic noise interferometry and volcanic tremor

Cross-correlation of the diffuse noise wave field measured at a pair of stations extracts coherent seismic energy arriving at both stations. Small perturbations to the arrival times of the phases in noise cross-correlation functions (NCFs) can then be measured to construct a temporal variation of the seismic velocity relative to a reference function (d*v*/*v*) (*11*). d*v*/*v* is usually estimated in the coda of the NCFs, a technique originally used in earthquake coda interferometry (*12*), as these signals have undergone significant scattering and so have sampled longer, denser paths within the medium of interest. This means that the coda is more sensitive to changes within that medium and so is less affected by spatial and temporal fluctuations of the noise source (*3*).

Most commonly, the noise sources used for ambient noise interferometry—as well as ambient noise tomography, which is an imaging method—are the highly energetic oceanic microseisms at periods of ~7 s (0.14 Hz) and 15 s (0.07 Hz). In those cases, the NCF for a given pair represents the seismic signal observed at one station as if there had been an impulsive source excitation at the other and vice versa. For a perfectly diffuse noise field, the NCF converges to a so-called Green’s function (*13*). For imaging studies, NCFs typically resemble surface waves that can be measured for absolute phase and group velocities between station pairs. However, an NCF with a clear surface wave is not a necessary requirement for passive monitoring of relative velocity changes (*14*–*16*). Rather, only a stable background noise structure, which repeatedly samples the medium in the same manner, is required. Then, stable NCFs with time can be constructed, and d*v*/*v* is measured from the very small changes within them.

In contrast, our approach makes use of a highly energetic volcanic tremor source (we filter between 0.33 and 1.0 Hz). We show that the volcanic tremor source is stable through time, and NCFs with consistent coda arrivals can be constructed. A previous study identified this tremor source at Kīlauea and showed how the tremor contaminates the attempt to reconstruct an interstation Green’s function (*17*). Our work reveals that the scattered NCF from the tremor source can be used to great effect to measure d*v*/*v*. Instead of an NCF representing energy that travels from one station to the other in a pair, we regard the NCF signal to represent consistent differential arrival times for phases traveling from the tremor source to each station of a pair (see Materials and Methods).

**Fig. 2:**
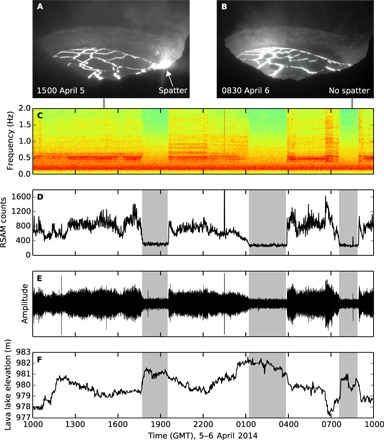
Volcanic tremor due to spatter. The lava lake fluctuates between spattering (white) and nonspattering regimes (gray). (**A**) Visible spatter source at the lake surface. (**B**) No spattering visible. (**C**) UWE amplitude spectrogram, low-pass–filtered at 10 Hz. The tremor associated with spatter has greatest amplitude just above 0.5 Hz. (**D**) Real-time seismic amplitude measurement (RSAM) at station UWE calculated by bandpass filtering between 0.33 and 5.0 Hz and then averaging seismic amplitude in 1-min intervals. (**E**) UWE seismic trace, bandpass-filtered between 0.33 and 5.0 Hz. (**F**) Lava lake elevation, generally observed to be a few meters higher during nonspattering compared to spattering regimes. (A), (B), and (F) reproduced with permission from Patrick *et al*. (*21*). GMT, Greenwich mean time.

**Fig. 3:**
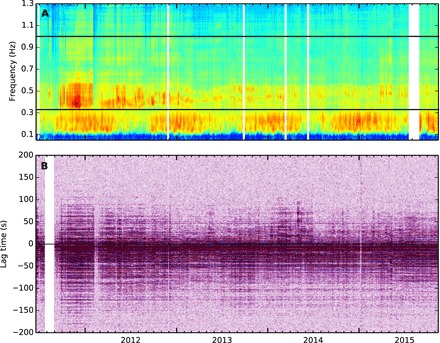
Stability of the noise source location. (**A**) Amplitude spectrogram for seismometer UWE (see Fig. 1 for location). Amplitude spectrograms are calculated in 10-min windows after decimating and low-pass filtering at 4 Hz. The median value is used for each day. The frequency band used in this study is bounded by black lines. White bands are data gaps. (**B**) NCFs stacked over 3-day moving windows for station pair PAUD-RIMD (see Fig. 1 for locations). The white band is a data gap.

The volcanic tremor source used in this study can be seen above ~0.3 Hz in the amplitude spectrogram for station Uwekahuna Vault (UWE) shown in Fig. 2C. This tremor, brightest at ~0.5 Hz, is associated with degassing and spattering of the lava lake surface (*10*, *18*–*21*). The lava lake is described as undergoing normal behavior during periods of spattering (Fig. 2A), associated with higher tremor amplitudes (Fig. 2, C to E) and gas emissions (*10*, *21*). During a nonspattering regime (Fig. 2B), tremor and gas emissions greatly decrease, and the level of the lake rises (Fig. 2F). Nonspattering phases typically last ~2 hours during the period of this study (*21*). This episodic behavior associated with the rise and fall of the lava lake surface (“gas pistoning”) results from gas bubbles accumulating near the top of the lake, followed by more efficient decoupling of gas from the lake (*21*). The tremor over the entire period of study can be seen in the amplitude spectrogram shown in Fig. 3A.

## RESULTS

### Stability of tremor source

Volcanic tremor can be seen above ~0.3 Hz in Fig. 3A, as well as the oceanic secondary microseism at ~0.1 to 0.3 Hz, displaying typical seasonal variation. Figure 3B demonstrates the ability to extract consistent NCFs with coherent coda arrivals in the frequency band (0.33 to 1.0 Hz) for almost the entire period of study. Coherent energy is seen in the coda of the NCF at negative lag times up to 120 s.

**Fig. 4:**
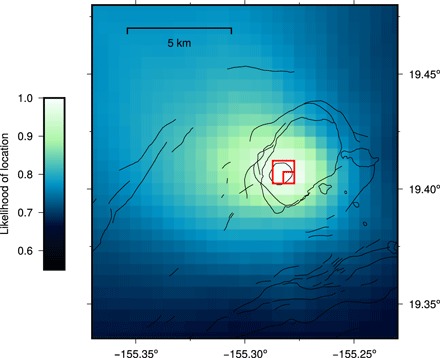
Tremor source location. See text for methodology. The most likely source location is shown by the small red square, which is also the closest grid point to the lava lake. The larger red square shows the results of a jackknife test: Of 1000 best locations, calculated with only half of the network (randomly chosen), 90% fall in this box.

We use the differential arrival time of the direct ballistic wave (first arrival in the NCF) to estimate the location of the tremor source in two-dimensional space and to investigate how stable this location is through time (*17*). Figure 4 shows that the most likely location of the noise source is in Halema’uma’u caldera at the closest grid point to the lava lake (small red square). We performed a jackknife test to investigate the effects of the network configuration on this location and to give an estimate of the error. The best source location was calculated 1000 times from the reference functions for randomly chosen network configurations using half the number of stations. More than 90% of results locate in the larger red square in Fig. 4 (approximately 1 km across). The robust estimate of location at or below Halema’uma’u caldera agrees with our suggestion that the source of tremor is spattering in the lava lake. The location has also been calculated from overlapping 10-day moving window stacks through the whole time period (June 2011 to November 2015) and is always found within the region of error (larger red square) in Fig. 4. This shows that the tremor source location is stable and validates the continuous use of this noise source. Source effects on our measurement of d*v*/*v* are still an important consideration, so we further examine this in Discussion.

### Correlation between d*v*/*v* and radial tilt on the time scale of days to weeks

**Fig. 5:**
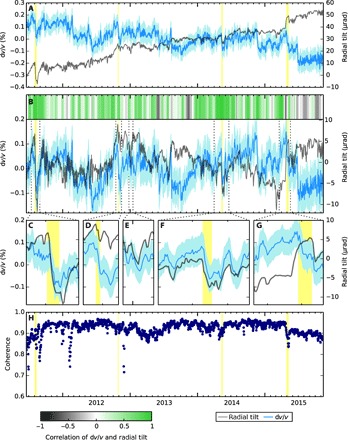
Results of d*v*/*v* and its relationship with radial tilt. (**A**) Raw relative velocity variations, d*v*/*v* (blue). Light blue shading indicates the error in the measurement, calculated from the linear regression of d*t* against *t*. Raw radial tilt measured at UWE (gray). The radial component of tilt is calculated with respect to the eruptive vent in Halema’uma’u caldera. (**B**) Short-term d*v*/*v* and radial tilt, estimated by linearly detrending the raw series. The gray-green bar shows the correlation coefficient between d*v*/*v* and radial tilt in 30-day moving windows with an overlap of 6 days. Times highlighted in yellow correspond to (from left to right) a breakout eruption at Puʻu ʻŌʻō (episode 60), a large deflation and an “anomalous” deflation event (see text), and an overflow of the lava lake onto Halema’uma’u caldera floor. (**C** to **G**) Enlargements of time periods in (B). (E) is a large DI event. (**H**) Coherence (correlation coefficient) between current 3-day moving window NCF and reference NCF.

A network-averaged relative velocity variation is found by taking the median d*v*/*v* of the pairs of the 22 closest stations from the eruptive vent (circled in Fig. 1; 231 pairs) and by stacking over 3-day moving windows. A linear trend from the raw d*v*/*v* time series in Fig. 5A (blue curve) is removed to estimate the short-term velocity variations shown in Fig. 5B.

Radial ground tilt, as recorded at site UWE (Fig. 5, gray curves), and the level of the lava lake both change in response to pressurization of the HMMR (*8*–*10*). An increase in UWE radial tilt corresponds to an inflation of Kīlauea summit and a rise in the level of the lava lake. We choose to study radial tilt here because tiltmeters offer better sensitivity and temporal resolution during DI events in comparison to other instrumentations (for example, GPS) at Kīlauea (*8*). The short-term variations in radial tilt have also been estimated by removing a linear trend from the raw time series (Fig. 5A).

A breakout eruption at Puʻu ʻŌʻō (episode 60), as highlighted in Fig. 5, began on 3 August 2011 and Puʻu ʻŌʻō drained and then refilled. A marked reduction and recovery of radial tilt at Kīlauea summit are also seen in d*v*/*v* (Fig. 5C). Figure 5 (D and E) shows large DI events as seen in the radial tilt and tracked by d*v*/*v*. d*v*/*v* also simultaneously drops during an “anomalous deflation event” in early May 2014 (Fig. 5F), as described by the Hawaiian Volcano Observatory (*22*). At this time, deflation was measured by tiltmeters at greater distances than for normal DI events, and seismicity was elevated in the summit region. In April 2015, the lava lake overflowed onto Halema’uma’u caldera floor. d*v*/*v* increased as Kīlauea inflated before the event but rapidly dropped when the lava lake overflowed and then never recovered to previous values (Fig. 5G).

**Fig. 6:**
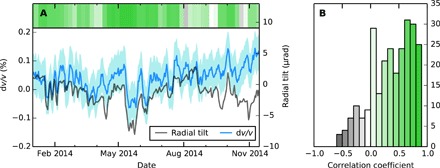
Evidence for the positive correlation between d*v*/*v* and radial tilt. (**A**) Example of period in 2014 when radial tilt and d*v*/*v* track each other closely (gray-green bar as in Fig. 5B). (**B**) Histogram of the correlation coefficients calculated between radial tilt and d*v*/*v* for 30-day moving windows with an overlap of 6 days over the whole time period, as shown in Fig. 5B.

The time series of radial tilt and d*v*/*v* are positively correlated in the short term (Fig. 5B). An increase in radial tilt (inflation) usually occurs simultaneously with an increase in d*v*/*v* (faster velocity) and vice versa. The gray-green bar in Fig. 5B shows the correlation coefficient between radial tilt and d*v*/*v* in 30-day-long moving windows, overlapping by 6 days. The coefficients are dominated by positive (green) values. A period that has a particularly clear positive correlation is shown in Fig. 6A. Many large V-shaped DI events can be seen in the radial tilt record, which are closely tracked by d*v*/*v*. In Fig. 6B, we show that the overlapping 30-day window correlation coefficients in Fig. 5B are skewed toward positive values, confirming the consistent positive correlation between radial tilt and d*v*/*v*. This correlation appears to end after the lava lake overflows in 2015.

### Correlation between d*v*/*v* and radial tilt on the time scale of years

The long-term variations in both radial tilt and d*v*/*v* can be seen in the raw time series (Fig. 5A). Radial tilt increases steadily in the long term from 2011 to 2015, suggesting overall inflation of the summit, whereas d*v*/*v* decreases. Vertical displacement measured by GPS at the summit also measures a long-term inflation (*23*). These raw time series of radial tilt and d*v*/*v* are anticorrelated over the 4-year period, with a cross-correlation coefficient of −0.73 at zero lag between them.

### Sensitivity of noise cross-correlation functions to changes in the medium

NCFs are sensitive to changes in both d*v*/*v* and the structure of the medium. The coherence (correlation coefficient) between the NCFs and the reference NCFs (Fig. 5H) can indicate whether the waveform has fundamentally changed, for example, because of a structural change in the medium (*3*). Coherence remains above 0.9 for almost the entire study period but drops temporarily during the breakout eruption at Puʻu ʻŌʻō in August 2011. During the overflow of the lava lake in April/May 2015, the coherence drops and does not recover to the same preceding value. This suggests that the complex NCFs containing many scattered phases have changed subtly because of a permanent change to the scatterers and reflectors in the edifice structure, presumably related to the addition of a layer of lava at the free surface. Therefore, d*v*/*v* and radial tilt may no longer correlate at this time because the reference NCF being used is not representative of the medium. The error in d*v*/*v* also permanently increases after the overflow. The decrease in coherence in February 2012 is roughly synchronous with a weakening of amplitude of the source, visible in Fig. 3 (A and B).

## DISCUSSION

We have shown a correlation between the time series of d*v*/*v* using noise interferometry and of surface deformation at a volcano. On the time scale of days to weeks, radial tilt and d*v*/*v* are positively correlated. We believe that this is the first time a correlation between d*v*/*v* and deformation has been found at this time scale consistently over a period of several years. In contrast, on the scale of years, radial tilt and d*v*/*v* are anticorrelated. Radial tilt at UWE is strongly correlated with the level of the lava lake (*9*), demonstrating a clear link between the magmatic system at Kīlauea and the summit deformation. During DI events, as the level of the lava lake rises, UWE radial tilt increases (and vice versa), resulting from pressure changes in the HMMR (*8*).

We propose that the changes in magma pressurization associated with the continual deflations and inflations of the summit also cause the changes in d*v*/*v*. When the magma pressurization increases, compression closes up cracks in the surrounding rock, and the elastic modulus and seismic velocity of the medium increase (*24*).

Several existing studies have also attributed seismic velocity changes to magma pressurization but have found the opposite trend, that is, seismic velocity decreases as a volcano inflates (*1*, *3*, *25*). Precursory seismic velocity drops were measured before several eruptions at Piton de la Fournaise volcano (*1*, *3*). Brenguier *et al*. (*1*) attributed this to an increase in magma pressurization causing dilatation and an opening of fractures at the edifice surface. Processes of pressurization due to heated and vaporized hydrothermal water have also been used to explain these short-term signals (*26*, *27*). At Merapi volcano in Indonesia, both increases and decreases in seismic velocity were measured during the same deformation events (*28*). The authors suggest that, because of topography and heterogeneity of Merapi volcano, both tensional and compressional stresses occur at different locations on the edifice. Similarly, for a deflation event at Miyakejima volcano, both velocity increases and decreases were detected (*29*). Velocity increases were attributed to deflation of two pressure sources, and velocity decreases were attributed to dilatation because of caldera collapse. Increases in seismic velocity were measured during inflation of a volcano, as seen in this study, at Merapi volcano before eruptions in 1992 (*30*) and 1998 (*31*). However, it has been suggested that these could be partly or entirely due to seasonal changes in groundwater level (*28*).

**Fig. 7:**
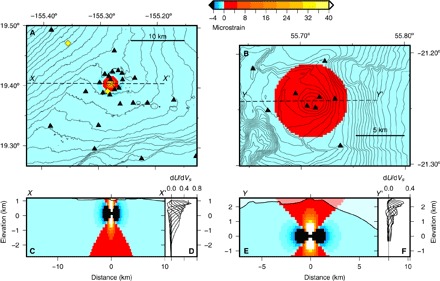
Model of volumetric strain due to inflation of point sources at 1-km depth (Kīlauea, left) and 2.6-km depth (Piton de la Fournaise, right). (**A**) Map view of volumetric strain model at Kīlauea for a deformation point source at 1-km depth below the surface. Seismic stations are depicted as black triangles, and strainmeters are depicted as yellow diamonds. Positive strain is extensional; negative strain is compressional. Note that black and white values lie below and above the limits of the color scale, respectively. (**B**) Map view of volumetric strain model at Piton de la Fournaise for a deformation point source at 2.6-km depth below the surface. Seismic stations (black triangles) were those used in the study by Brenguier *et al*. (*1*). (**C**) Cross section of strain model for the dashed line shown in (A). Surface topography is marked on, but the model was calculated for an elastic half-space. (**D**) Rayleigh wave depth sensitivity kernels at Kīlauea for frequencies between 0.33 and 1.0 Hz (periods, 1 to 3 s every 0.25 s). (**E**) Same as in (C) but corresponding to Piton de la Fournaise and (B). (**F**) Same as in (D) but for Piton de la Fournaise.

We suggest a model based on strain theory at volcanoes to reconcile some of these observations (Fig. 7). The pattern of deformation at a volcano due to a deforming source greatly depends on the depth of that source; a deeper source results in a greater region in the shallow subsurface undergoing extension. We assume that a change in velocity is proportional to a change in strain (*29*, *32*–*34*). For a point pressure source at depth *d*, the sign of the strain field changes at a distance *r* = *d*√2 at the surface of the elastic half-space (that is, neglecting topography) (*35*). When a pressure source is inflating, an area directly above the source undergoes extensional strain, whereas the surrounding area undergoes compressional strain. The deeper the source, the larger the area of extension. At depth, volumetric strain is extensional above and below the source and compressional at the sides (Fig. 7, C and E). We suggest that many results that detect a decrease in d*v*/*v* as a volcano inflates are dominated by the extensional strain and the associated opening of pores and cracks. The pressure source at Kīlauea, which is assumed to be the HMMR (*8*), is relatively shallow. Estimates of the depth include ~1 km (*36*), 1.6 km (*37*), and 1 to 2 km (*7*). There are two borehole strainmeters at Kīlauea: MLS, 11 km northwest of the summit vent; and KWL (now offline), 1.5 km south of the vent (Fig. 1). According to Anderson *et al*. (*8*), MLS detects larger DI deflations as extensional strain and inflations as contractional strain. Unfortunately, KWL did not accurately record strain amplitude, although the sign was probably correct (*8*). KWL also recorded contraction during inflation and vice versa. The sign of strain from both these instruments agrees with the measured sign of d*v*/*v*, that is, when the strainmeters measure contraction, d*v*/*v* increases. Assuming that the HMMR is reasonably approximated by a point source and the sign of KWL is correct, a maximum constraint on the depth of the HMMR is just over 1 km; this is the depth that we use in the rest of this discussion. Using this simple strain model, the radius of the area of extensional strain is approximately 1.4 km, so a large part of Kīlauea summit undergoes contraction (Fig. 7, A and C). We have calculated depth sensitivity kernels for Rayleigh waves for our frequency band using 1D velocity models (Fig. 7, D and F) (*38*, *39*). Because the surface waves at Kīlauea are likely to be most sensitive at ~0.5 to 1.5 km below the surface, even in the region of extension at the surface, the NCFs are still likely to be sensitive to contraction at depth (see Fig. 7, C and D). It is this field of contraction during inflations (and vice versa) that we believe dominates our measurement of d*v*/*v*.

Our theory can also be compared to an existing study at Piton de la Fournaise volcano. Obermann *et al*. (*3*) used a least-squares inversion to map d*v*/*v* in space for an inflation before an eruption. In addition to a central area of velocity decrease (during inflation before an eruption), the authors also measure a velocity increase at a greater distance from the summit. For a deformation source at ~2.6-km depth below the surface (*40*), we would expect the inner region of extension and hence velocity decrease to be over 7.4 km in diameter. This is slightly smaller than the radius at which the velocity changes from a decrease to an increase (*3*), but is in reasonable agreement considering that this estimate assumes a deforming point source and neglects topography. These results suggest that our simple model based on strain theory could be a good starting point for understanding velocity changes at volcanoes. This gains support from our demonstration that deformation caused by magma pressurization controls d*v*/*v*, with such an excellent correlation between the two geophysical observables. Measured values of d*v*/*v* are consistent with expected values as estimated from published strain data at MLS, which are described further in the Supplementary Materials and following the method of Hotovec-Ellis *et al*. (*32*, *33*).

To interpret changes in the measured d*v*/*v*, we assume that they are dominated by changes in the medium rather than by source effects. Coda waves result from multiple scattering of seismic waves in a heterogeneous medium. Because coda waves propagate much further than direct waves, they are more sensitive to changes in the medium and less sensitive to changes in the noise source (*3*). Nevertheless, it is worth further investigating the idea that changes in the elevation of the tremor source are affecting our measurement of d*v*/*v*, because the surface of the lava lake—where the spatter occurs—is inherently linked to the inflation and radial tilt of the summit.

We suggest that d*v*/*v* is a real measurement of velocity in this study and is not controlled by vertical motion of the tremor source for the following further reasons. First, we believe that the NCFs are dominated by surface waves because we measure greatest amplitudes in the NCFs at time lags corresponding to arrivals traveling at 1 km/s from the tremor location. Many existing studies assume that the wave field originating from the oceanic microseisms is dominated by surface waves (*1*, *41*). However, a wave field resulting from tremor associated with a spattering lava lake is less well understood. Nevertheless, the phases will be of very long wavelengths (~1 to 9 km) at these frequencies. Because depth changes of the lava lake are, at most, 50 m for the largest DI events (*8*), this vertical movement is unlikely to affect the NCFs. Second, we believe that, if vertical movement of the tremor source affected d*v*/*v* by changing the relative path lengths between the source and stations, which would be relevant if body waves make up a significant part of the NCF (*42*), we would measure the opposite trend in d*v*/*v*. Let us consider a pair of stations on the surface and limiting cases of the tremor source at the surface and infinite depth. The differential interstation distance (see Materials and Methods) is at a maximum when the tremor source is at the surface and tends to zero for a tremor source getting infinitely deep. Therefore, shallow and deep tremor sources correspond to measurements of the slowest seismic velocity and an infinitely fast seismic velocity, respectively. This trend is opposite to the measured trend of faster velocity at a higher elevation of the lava lake (that is, a shallow tremor source). We thus conclude that our measurements of d*v*/*v* reflect real changes in seismic velocity and are not spuriously produced by vertical movement of the tremor source in the conduit.

The result of d*v*/*v* correlating with radial tilt is consistent when measured at different time lags in the NCFs, when using a window length of at least 20 s on both sides of the NCFs and when averaged over many pairs. Given that some stations lie in the inner region of strain suggested in our model, one might expect to measure a d*v*/*v* time series that negatively correlates with radial tilt using these stations. Further, the magnitude of strain change is greater within the inner region than in the outer, so we suggest testing this theory at other volcanoes with a shallow deformation source. However, we do not find this negative correlation at Kīlauea and we suggest the following two reasons for this. First, we believe that the coda of the NCFs is sensitive to a wide area extending outside the inner region of strain because of scattering of the seismic waves. It is difficult to avoid measuring the coda; limiting measurements to the early ballistic arrivals means that a short window length for the frequency of study must be used, and we then measure a noisy time series. We further note that the area of extension at the surface is also undergoing compression at depth (for an inflation, see Fig. 7C). Therefore, even phases—probably surface waves, as suggested above—traveling in the region of near-surface extension could be sensitive to the compression at depth (Fig. 7D). Second, if we limit our average of d*v*/*v* to the stations closest to the source, there are very few pairs. We find that time series become much noisier when the number of pairs decreases, and ideally, a minimum of ~40 pairs is needed. However, when we include more stations to increase the number of pairs, we start to sample the outer strain region.

Because the long-term trends of radial tilt and d*v*/*v* are oppositely correlated to those in the short term, the same mechanism cannot be at play. The source of long-term inflation could be deeper than the HMMR, and so a greater area of extensional strain would exist at the surface. If the summit reservoir at 3- to 5-km depth (*7*) inflates, then extension could be seen over an area with a radius as large as 7 km. Another suggestion is that inflation of the summit could be accompanied by an increase in the presence of magma and/or hydrothermal fluids within the pore spaces of the summit (*43*). It is also possible that the seaward motion of Kīlauea’s south flank results in crust relaxation and opening of the pore space. Flank movement has been suggested to account for a velocity drop measured at Piton de la Fournaise before an eruption in April 2007 (*44*).

To our knowledge, we have shown for the first time a consistent correlation between a daily time series of relative velocity, d*v*/*v*, and daily surface deformation measurements. Having a reliable record of d*v*/*v* at this time scale is an important step forward in terms of volcano monitoring with noise interferometry. This result also provides an opportunity to understand better the dominant mechanism controlling the seismic velocities at volcanoes, which has been difficult in previous work, particularly during intereruptive periods. The clear link with deformation associated with DI events suggests that pressurization of the shallow reservoir at Kīlauea summit is also affecting seismic velocity across the summit region.

## MATERIALS AND METHODS

### Seismic network

The seismometers used in this study are maintained by the Hawaiian Volcano Observatory, U.S. Geological Survey. Twenty-seven instruments around Kīlauea summit were used: 16 broadband and 11 short-period instruments. All stations mentioned in this paper are broadband: PAUD (T120, 120-s corner), RIMD (T120, 120-s corner), and UWE (STS2, 120-s corner).

### Seismic noise interferometry to measure d*v*/*v*

We used the freely available program MSNoise to measure d*v*/*v* (*45*). The data were stored in day-long, 100-Hz miniSEED files. Only vertical components were used. The data from each station were preprocessed individually. An initial bandpass was applied (0.01 to 8.0 Hz), and the waveforms were demeaned, tapered, and downsampled to 20 Hz. The waveforms were then temporally normalized (by clipping at the root mean square multiplied by 1.5) and spectrally whitened in 30-min windows (*46*).

NCFs were calculated for every pair in the network in 30-min windows for time lags of ±120 s. The NCFs were stacked for each day, and then the daily NCFs were stacked over 3-day moving windows. A reference NCF was also calculated for each pair by stacking the daily NCFs over the whole period.

The Moving-Window Cross-Spectral (MWCS) method, also known as the doublet method, was used to measure the dephasing between the NCF and the reference NCF through time (*47*). The MWCS method works in the frequency domain and measures a delay time (d*t*) at different lag times (*t*) in the current NCF, relative to the reference NCF. Only points with an error of less than 0.1 s, a coherence of more than 0.65 and a d*t* of less than 0.1 s were accepted. A weighted linear regression was calculated in a 30-s window in the NCF to calculate d*t*/*t*. The minimum time lag of this window was chosen by dividing the differential interstation distance from the tremor source by a velocity of 0.8 km/s. The differential interstation distance from the source for a pair A-B refers to

distance(source→A)− distance(source→B)

This is shown diagrammatically in the Supplementary Materials. This distance is required because the source of seismic energy is not external and isotropic to the network. A velocity of 0.8 km/s—slower than the ballistic arrivals in the NCF—allows measurement of the coda of the waves. This technique was originally used in earthquake coda interferometry (*12*). Then, the relative velocity change was calculated using

$$\frac{dv}{v}=-\frac{dt}{t}$$ (1)

assuming a homogeneous relative velocity change (*30*). We averaged d*v*/*v* for the closest 22 stations from the source (231 pairs). We rejected a pair’s results for a day if the error from the linear regression of d*t* against *t* is greater than 0.01%.

It is possible to spuriously measure apparent d*v*/*v* variations because of a change in frequency content of the noise source (*48*) using the “stretching technique” (*11*). The MWCS method, in contrast, is theoretically relatively unaffected by this problem because the amplitude spectrum and phase spectrum are separated before making the measurements. Further discussion of possible changes in frequency content affecting the measurement of d*v*/*v* can be found in the Supplementary Materials.

### Tremor source location method

The NCFs are asymmetrical because the noise wave field is not isotropic, but it mainly originates in one location. We exploit this fact to locate the noise source, following the method of Ballmer *et al*. (*17*). We constructed a 0.005° (c. 500 m) geographical grid and considered each point to be a potential source location. Arrival times were estimated in the NCFs for each pair of stations for each grid point, assuming a lateral propagation velocity of 1 km/s. By testing velocities of 0.5 to 3.3 km/s, we find that the estimate of source location is relatively insensitive to the assumed velocity. The total absolute amplitudes in 4-s windows around the expected arrival times in the NCFs (using the differential interstation distance) for each pair were added together. This then represents the likelihood of the source being located at that grid point. The estimate of source location in Fig. 4 was calculated from the reference NCFs for all the pairs.

### Strain modeling

Strain modeling used routines from the freely available package Coulomb 3.3 (*49*, *50*). Depth sensitivity kernels were calculated with the program surf96 (*51*) and published velocity models (*38*, *39*).

## Supplementary Material

http://advances.sciencemag.org/cgi/content/full/3/6/e1700219/DC1

## SUPPLEMENTARY MATERIALS

Supplementary material for this article is available at http://advances.sciencemag.org/cgi/content/full/3/6/e1700219/DC1 Differential interstation distance from source Coda arrivals in NCFs Two reference functions Expected change in d*v*/*v* from strain data Frequency variations in the volcanic tremor source d*v*/*v* measured between 0.1 and 0.3 Hz Robustness of positive correlation between radial tilt and d*v*/*v* and comparison with meteorological effects and seismicity fig. S1. Explanation of differential interstation distance from source. fig. S2. Decay of coherent coda wave arrivals in the NCFs. fig. S3. Results when using two reference functions. fig. S4. Detailed view of frequency content of the volcanic tremor source and d*v*/*v*. fig. S5. Comparison of d*v*/*v* with 0.33- to 1-Hz and 0.1- to 0.3-Hz filters. fig. S6. Radial tilt-d*v*/*v* correlation and its association with meteorological effects and seismicity.
