# Supplementary material for: Relative seismic velocity variations correlate with deformation at Kīlauea volcano
Source: Sci Adv. 2017 Jun 28;3(6):e1700219. doi: 10.1126/sciadv.1700219 (PMC5489268; doi:10.1126/sciadv.1700219)
Supplement: http://advances.sciencemag.org/cgi/content/full/3/6/e1700219/DC1 [file supp_3_6_e1700219__index.html]

Science Advances | Science Advances

## Supplementary Materials

**This PDF file includes:**

- Differential interstation distance from source
- Coda arrivals in NCFs
- Two reference functions
- Expected change in d*v*/*v* from strain data
- Frequency variations in the volcanic tremor source
- d*v*/*v* measured between 0.1 and 0.3 Hz
- Robustness of positive correlation between radial tilt and d*v*/*v* and comparison with meteorological effects and seismicity
- fig. S1. Explanation of differential interstation distance from source.
- fig. S2. Decay of coherent coda wave arrivals in the NCFs.
- fig. S3. Results when using two reference functions.
- fig. S4. Detailed view of frequency content of the volcanic tremor source and d*v*/*v*.
- fig. S5. Comparison of d*v*/*v* with 0.33- to 1-Hz and 0.1- to 0.3-Hz filters.
- fig. S6. Radial tilt-d*v*/*v* correlation and its association with meteorological effects and seismicity.

Download PDF

**Files in this Data Supplement:**

- Adobe PDF - 1700219\_SM.pdf
