# Supplementary material for: Relative seismic velocity variations correlate with deformation at Kīlauea volcano
Source: Sci Adv. 2017 Jun 28;3(6):e1700219. doi: 10.1126/sciadv.1700219 (PMC5489268; doi:10.1126/sciadv.1700219)
Supplement: http://advances.sciencemag.org/cgi/content/full/3/6/e1700219/DC1 [file 1700219_SM.pdf]

Science Advances

AAAS

advances.sciencemag.org/cgi/content/full/3/6/e1700219/DC1

# Supplementary Materials for

## Relative seismic velocity variations correlate with deformation at Kīlauea volcano

Clare Donaldson, Corentin Caudron, Robert G. Green, Weston A. Thelen, Robert S. White

Published 28 June 2017, *Sci. Adv.* **3**, e1700219 (2017)
DOI: 10.1126/sciadv.1700219

**This PDF file includes:**

- Differential interstation distance from source
- Coda arrivals in NCFs
- Two reference functions
- Expected change in d*v*/*v* from strain data
- Frequency variations in the volcanic tremor source
- d*v*/*v* measured between 0.1 and 0.3 Hz
- Robustness of positive correlation between radial tilt and d*v*/*v* and comparison with meteorological effects and seismicity
- fig. S1. Explanation of differential interstation distance from source.
- fig. S2. Decay of coherent coda wave arrivals in the NCFs.
- fig. S3. Results when using two reference functions.
- fig. S4. Detailed view of frequency content of the volcanic tremor source and d*v*/*v*.
- fig. S5. Comparison of d*v*/*v* with 0.33- to 1-Hz and 0.1- to 0.3-Hz filters.
- fig. S6. Radial tilt-d*v*/*v* correlation and its association with meteorological effects and seismicity.

## Supplementary Materials

### Differential interstation distance from source

When a noise source is located inside the seismic network, the interstation distance for a pair of stations is less relevant when considering the expected arrival times of phases in the Noise Correlation Functions (NCFs). Instead we use the differential interstation distance, for example, when locating the noise source and when deciding on a window in the NCFs to use to calculate dt/t and dv/v. Figure S1 shows how we define both this distance and the window in the NCFs that are used in this study.

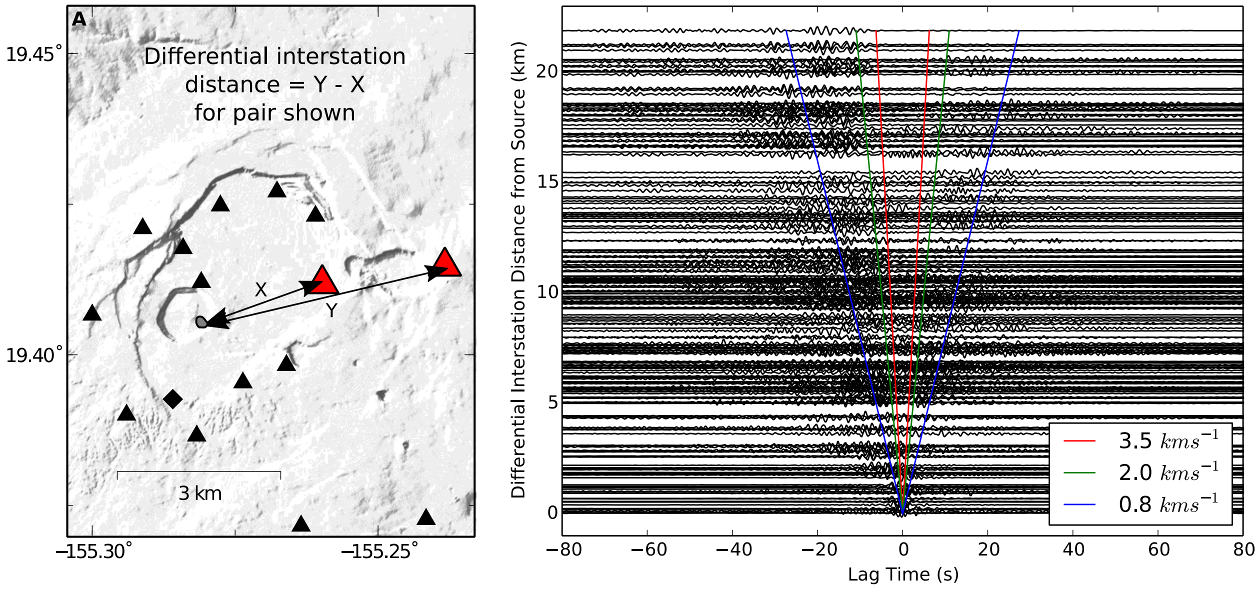


**fig. S1. Explanation of differential interstation distance from source.** (**A**) For each pair we define the differential interstation distance from the source as Y-X. (**B**) Move-out plot for reference functions of station pairs, with differential interstation distance from the source for each pair on the y-axis. For distances < 5 km, only a quarter of pairs (randomly chosen) are shown, to aid visual interpretation. To calculate dt/t and dv/v we use 30 s windows in the NCFs with a minimum time lag defined by a velocity of 0.8 km/s (blue line).

### Coda arrivals in NCFs

To check that the coda arrivals in our NCFs are related to the scattering of seismic waves between two stations, as we suggest in this study, the log of the envelope of amplitude in a NCF against lag time is plotted in fig. S2. As expected, the log of the envelope of amplitude of coda arrivals in the NCFs decays linearly before reaching a plateau.

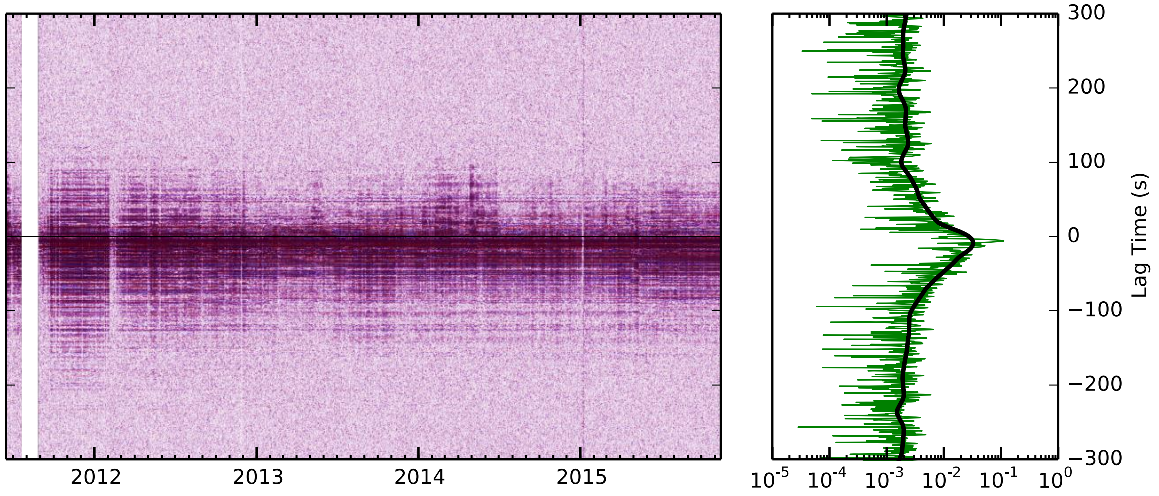


**fig. S2. Decay of coherent coda wave arrivals in the NCFs.** (**A**) 3-day stacks of noise cross-correlation functions (NCFs) for pair PAUD-RIMD, as shown in Fig. 2A, but to greater time lags of +/- 300 s. (**B**) Green line shows the envelope of amplitude of the 3-day stack NCF for 2014-06-01. Black line shows a smoothed form.

## Two reference functions

As discussed in Results, the lava lake overflowed onto Halema'uma'u caldera floor on April 28th 2015 for several days. The coherence (correlation coefficient) between the current and reference NCFs dropped instantaneously with the overflow and never recovered to previous values. The error in dv/v increased and the correlation between dv/v and radial tilt also deteriorated. This suggests that the medium changed and the reference function may be unrepresentative for April 28th 2015 onwards. We test this idea further by computing dv/v using two reference functions: one before and after the lava lake overflow (fig. S3). The new reference function for April 28th 2015 onwards results in an improvement in the correlation between dv/v and radial tilt from previously. There remains a large decrease in dv/v in late June 2015 which is not seen in radial tilt; the reason for this is unclear, not obviously being related to either a change in the tremor source, a volcanic event, or significant meteorological events.

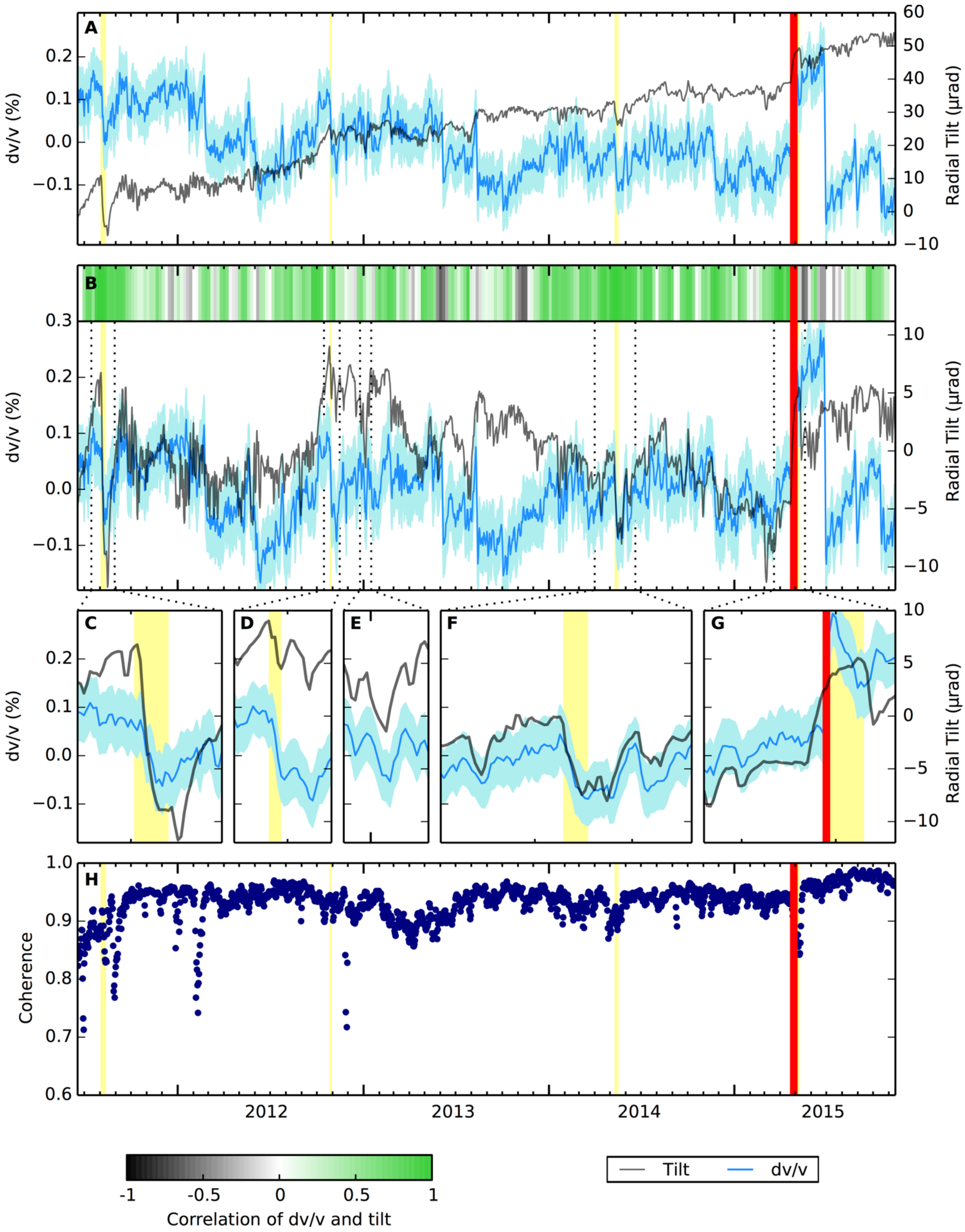


**fig. S3. Results when using two reference functions.** Results as in Fig. 5, but with two reference functions: one is a linear stack of all days up until April 27th 2015, before the lava lake overflowed, the other from April 28th 2015 (thick red line) onwards. Note that comparison of the magnitude of dv/v variations between the two time periods is invalid as they are now relative to separate and

arbitrary references. (**A**) Raw relative velocity variations, dv/v (blue). Light blue shading indicates the error in the measurement, calculated from the linear regression of dt against t. Raw radial tilt measured at UWE (gray). The radial component of tilt is calculated with respect to the eruptive vent in Halema'uma'u caldera. (**B**) Short-term dv/v and radial tilt, estimated by linearly detrending the raw series. The gray-green bar shows the correlation coefficient between dv/v and radial tilt in 30-day moving windows with an overlap of 6 days. Times highlighted in yellow correspond to (from left to right): a breakout eruption at Pu‘u ‘Ō‘ō (Episode 60); a large deflation and an ‘anomalous’ deflation event (see text); and overflow of the lava lake onto Halema'uma'u caldera floor. (**C-G**) Enlargements of time periods in B. E is a large deflation-inflation event. (**H**) Coherence (correlation coefficient) between current 3-day moving window NCF and reference NCF.

## Expected change in d*v/v* from strain data

To test whether the changes in strain detected at MLS could account for our measurements of dv/v, we follow the work of Hotovec-Ellis *et al.* (*28*). Relative velocity change is proportional to strain, the shear modulus and two of the three Murnaghan third-order elastic constants (Equation 9 in Hotovec-Ellis et *al.* (*28*)). For a characteristic change in strain measured at MLS for a deflation-inflation event, we refer to Fig. 11.2 in Anderson *et al.* (*8*). Over the event, the strain changes by ~100 nstrain. Using a value for ($m / \mu$) of $-1 \times 10^{-4}$ and assuming $n$ to be approximately equal to $m$, as do Hotovec-Ellis *et al.*, we would expect this change in strain to correspond to a dv/v of 0.05 %. The change in radial tilt at station UWE for the same event is ~8 μrad. Although variable, we generally find that a change of 30 μrad corresponds to a change in dv/v of approximately 0.55 %, by comparing scales in Fig. 3B. Therefore, we would expect to have measured a change in dv/v of 0.14 % for this DI event. This is larger than the theoretical estimate of 0.05%. However, since the strainmeter is 11 km from the summit vent and larger changes in strain would be expected nearer the deforming source (Fig. 6A), we would expect to measure a greater change in velocity than predicted from strain measured at this instrument. Although basic, this calculation is a helpful validation of our hypothesis that strain theory can help explain observed changes in seismic velocity at volcanoes.

## Frequency variations in the volcanic tremor source

A possible source effect which could affect our measurement of dv/v is the frequency content of the seismic waves generated from the noise source (*46*) . If lava lake spattering produces surface waves by beating on the conduit walls, the height of the lava column may directly affect the frequency of those waves. However, the methodology of noise interferometry should not be affected by such a change. Firstly, before cross-correlation, the seismograms are spectrally whitened to flatten out the amplitude in the frequency domain. Secondly, the Moving-Window Cross-Spectral method (MWCS, see Materials and Methods) is theoretically relatively unaffected by frequency variations because the

amplitude spectrum and phase spectrum are separated before making the measurements (*46*). As an additional check, we plot a detailed spectrogram and compare this to dv/v, for a 50-day period in 2014 (fig. S4). Changes in the amplitude and frequency of the tremor are visible but there is no obvious systematic relationship with dv/v. This may nevertheless be a sensible target for future work; the sensitivity of interferometry to frequency content is important for the field as a whole.

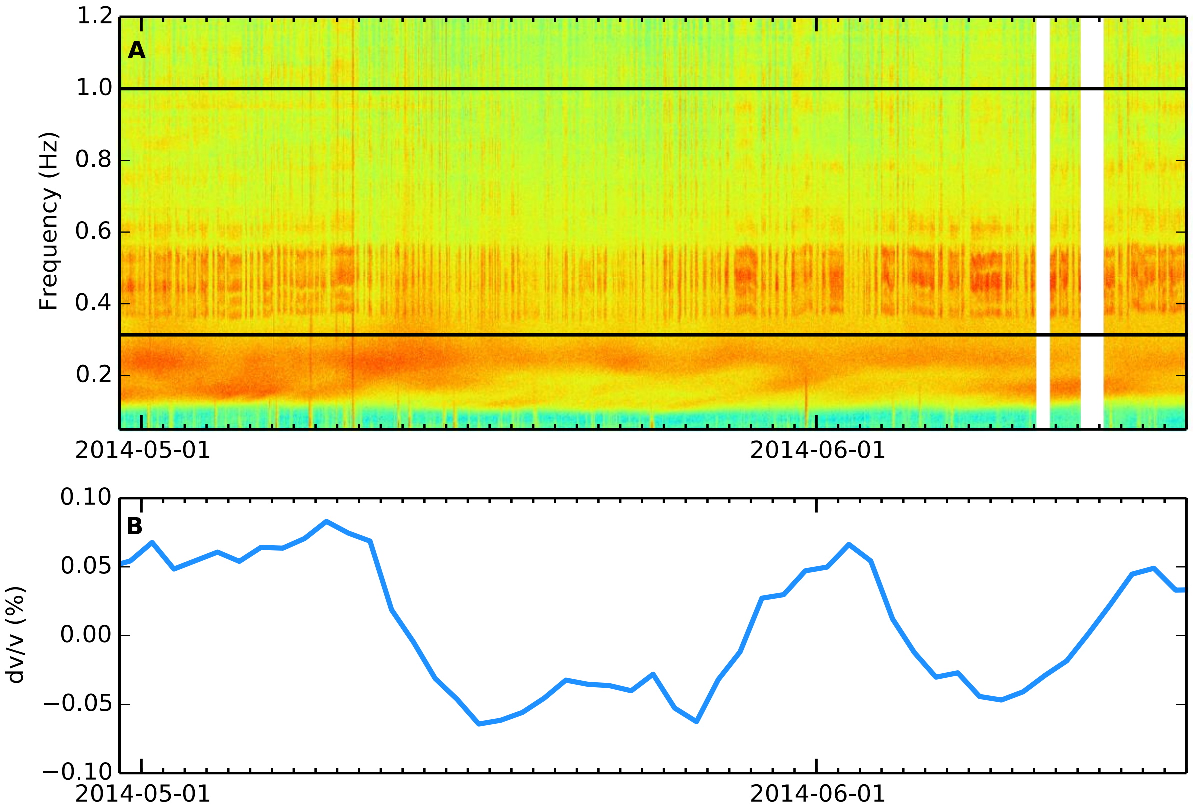


**fig. S4. Detailed view of frequency content of the volcanic tremor source and dv/v.** (**A**) Amplitude spectrogram for station UWE calculated in 10-minute windows after decimating and band-pass filtering between 0.05 – 4.0 Hz. Black horizontal lines show the frequency band used in measurement of dv/v (0.33 – 1.0 Hz). (**B**) dv/v as shown in Fig. 5 for 50 days in 2014.

## dv/v measured between 0.1 and 0.3 Hz

Use of the volcanic tremor source at Kīlauea has resulted in a seemingly high-quality time series of dv/v. We compare this to results calculated when filtering out the tremor source in fig. S5 (red curves). dv/v is measured in the frequency band 0.1 – 0.3 Hz using stations evenly spread across the large region shown in Fig. 1. We do not use all the stations close to Kīlauea caldera because of possible spatial aliasing effects at these longer wavelengths. The 0.1 – 0.3 Hz results require much longer stack lengths to gain an interpretable time series, perhaps suggesting that the NCFs are less successful at illuminating the medium with the oceanic microseisms at these frequencies. They also

show little similarity with the 0.33 – 1.0 Hz results, possibly because the NCFs are sensitive to different depths at longer periods.

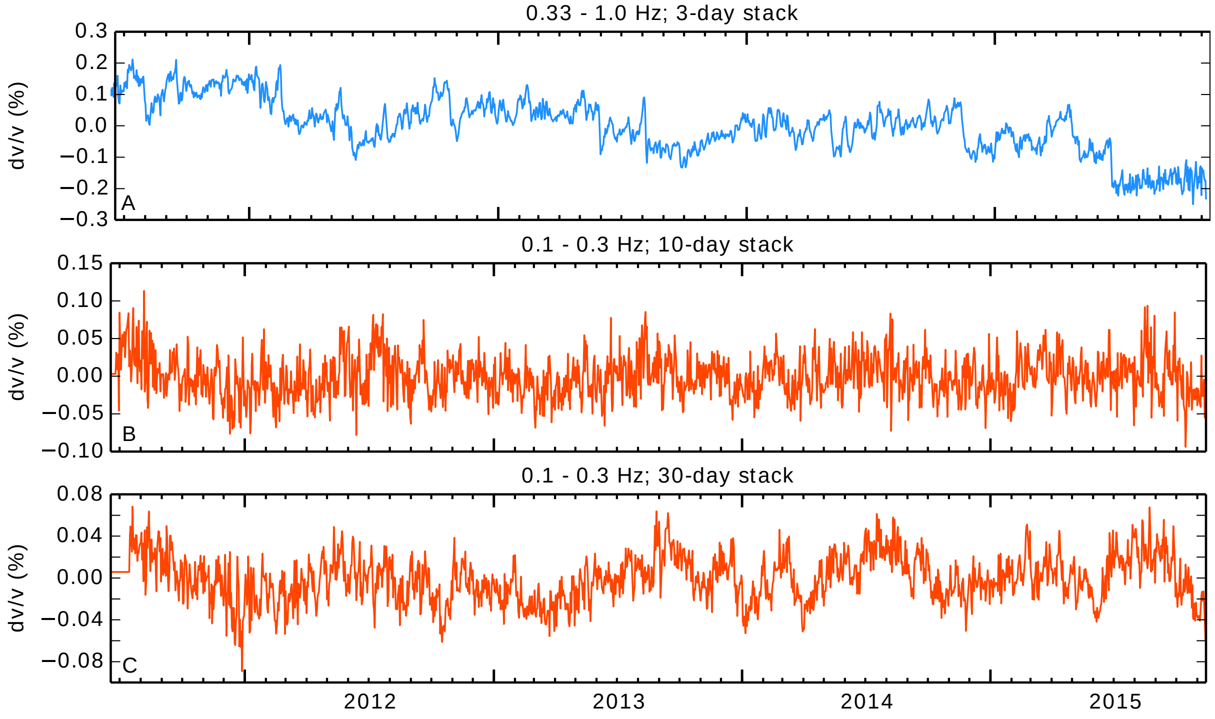


**fig. S5. Comparison of dv/*v* with 0.33- to 1-Hz and 0.1- to 0.3-Hz filters.** (**A**) Relative velocity variations (dv/v) as shown in Fig. 5, for the frequency band 0.33 – 1.0 Hz, 3-day moving window stack. (**B**) dv/v measured in the frequency band 0.1 – 0.3 Hz, 10-day stack, averaged for all pairs using 14 stations over Kīlauea (AHUD, AIND, DESD, DEVL, HLPD, HTCD, KAED, KNHD, MITD, MLOD, PAUD, POLD, UWE, RSDD). (**C**) dv/v as in B but a 30-day stack.

## Robustness of positive correlation between radial tilt and dv/*v* and comparison with meteorological effects and seismicity

In Results, we show the correlation coefficient between radial tilt at UWE and dv/v for 30-day moving windows with a 6-day overlap for the duration of the study. In fig. S6 we further investigate the correlation over different window lengths. The 30-day window is representative of the correlation between the short-term variations in radial tilt and dv/v that are the focus of this study. Times of poor correlation can be seen across all five gray-green bars. These are not obviously related to meteorological or volcanic events as seen in the precipitation, pressure and number of earthquakes at Kīlauea.

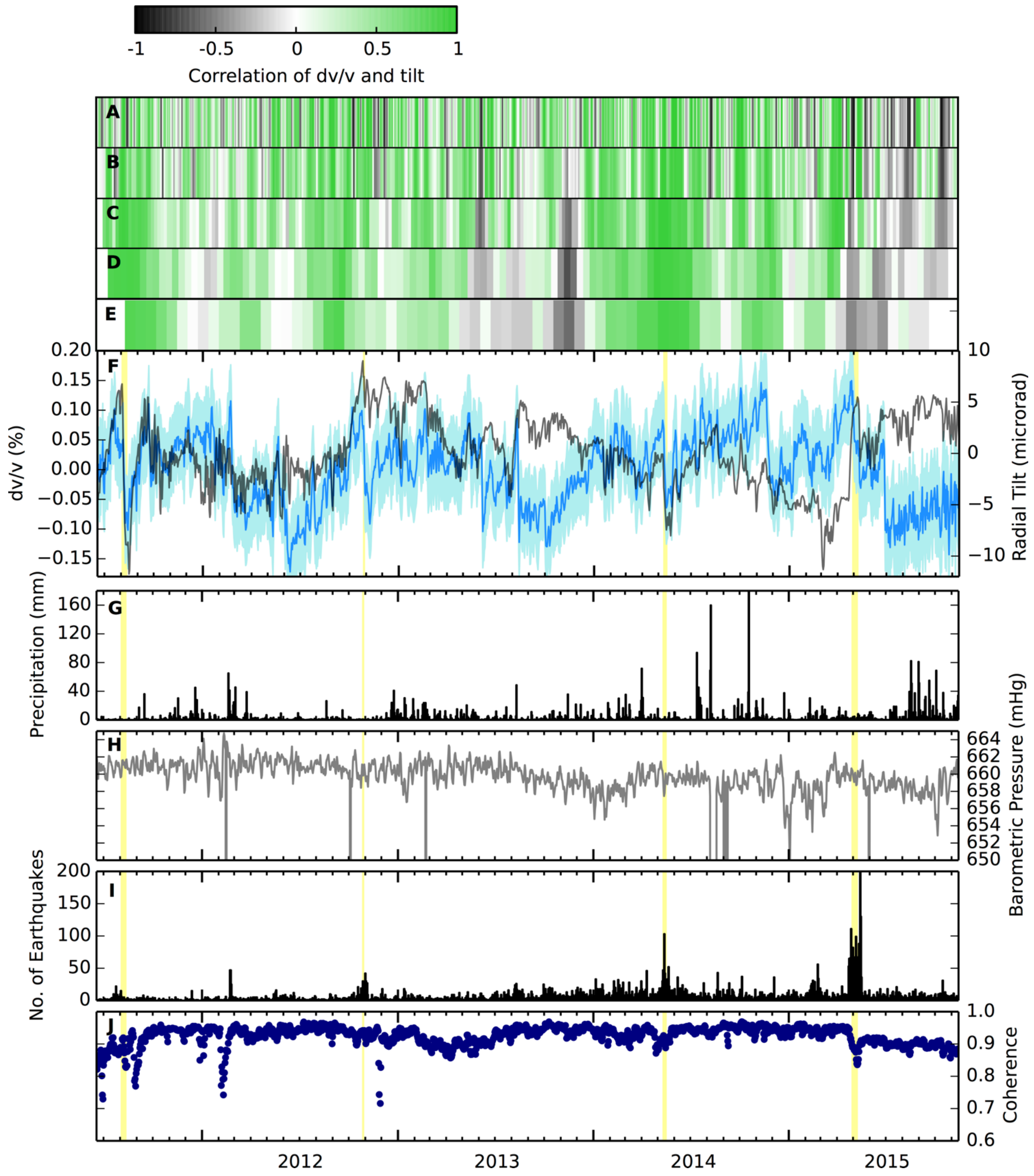


**fig. S6. Radial tilt-d*v*/*v* correlation and its association with meteorological effects and seismicity.** (**A**)-(**E**) Correlation between UWE radial tilt and dv/v over 7, 15, 30, 60 and 100-day window lengths. The moving windows overlap by one fifth of the window length (e.g. 6 days of overlap for 30-day window). (**F**) Short-term variations in dv/v (blue) and radial tilt (gray) as in Fig. 5B. (**G**) Precipitation and (**H**) barometric pressure measured at Hawaii Volcanoes National Park – Observatory weather station, averaged per day. This data is available online from the U.S. Department of the Interior National Park Service. (**I**) Earthquakes around Kīlauea recorded by the Hawaiian Volcano Observatory with 8 or more stations. (**J**) Coherence (correlation coefficient) between the current and reference NCFs.
